# Cerebral oxygen saturation after multiple perioperative influential factors predicts the occurrence of postoperative cognitive dysfunction

**DOI:** 10.1186/s12871-015-0117-6

**Published:** 2015-10-26

**Authors:** Cheng Ni, Ting Xu, Nan Li, Yang Tian, Yongzheng Han, Qingsheng Xue, Min Li, Xiangyang Guo

**Affiliations:** 1 Department of Anesthesiology, Peking University Third Hospital, Beijing, 100191 China; 2 Research Center of Clinical Epidemiology, Peking University Third Hospital, Beijing, 100191 China; 3 Department of Anesthesiology, Ruijin Hospital, Shanghai Jiaotong University School of Medicine, Shanghai, 200025 China

**Keywords:** Cerebral oxygen saturation, Postoperative cognitive dysfunction, Elderly patient, Total knee arthroplasty, Perioperative influential factors

## Abstract

**Background:**

Postoperative cognitive dysfunction (POCD) is a frequent complication in elderly patients undergoing major non-cardiac surgery, but its etiology is still unclear. Cerebral oxygen saturation (ScO_2_) represents the balance of cerebral oxygen supply and demand. The aim of present study was to evaluate the relationship between perioperative ScO_2_ and POCD, and to verify the hypothesis that the value of ScO_2_ after multiple perioperative influential factors could predict POCD in elderly patients undergoing total knee arthroplasty (TKA).

**Methods:**

Seventy eight Patients aged more than 65 years undergoing elective TKA with intrathecal anesthesia were enrolled. Cognitive functions were assessed one day before and 6 days after surgery, and POCD were defined according to ISPOCD. Demographics were recorded. Perioperative ScO_2_, blood pressure (BP), blood gas analysis and other clinical data were monitored and recorded, then the decrease of ScO_2_, BP and PaO_2_ after influential factors were calculated.

**Results:**

POCD occurred in 15 patients (19.2 %). BP decreased after anesthesia induction and tourniquet deflation, and PaO_2_ decreased after cement implantation, then percentage decrease of BP was higher in POCD group. ScO_2_ of POCD group is significantly lower than non-POCD group (*P* < 0.05), and the absolute value and percentage decrease of ScO_2_ became significant between two groups after multiple influential factors. ScO_2_ after all influential factors (anesthesia induction, cement implantation and tourniquet deflation) had the best predictive performance for POCD (AUC = 0.742), and the optimal threshold was 66.5 %.

**Conclusions:**

Perioperative ScO_2_ of patients with POCD is lower than patients without POCD. ScO_2_ after multiple perioperative influential factors could be an effective predictor for POCD, which reveal an important role of ScO_2_ decrease in the development of POCD and provide possible treatment target.

## Background

Cerebral oxygen saturation (ScO_2_) represents mixed oxygen saturation of cerebral arterial and venous (25 and 75 %) [1], and provides the status of cerebral hemodynamics in multiple pathological processes [2, 3]. The level of ScO_2_ depends on the balance of cerebral oxygen supply and demand. Cerebral perfusion, arterial oxygen pressure and hemoglobin concentration affect cerebral oxygen supply [4], while anesthetic depth and body temperature affect cerebral oxygen demand [5]. In cardiac surgery, ScO_2_ decrease associates with cardiopulmonary bypass cannula malposition [6] and postoperative stroke [7], and optimizing cerebral oxygen delivery under ScO_2_ monitoring reduces the risk of stroke [8]. Furthermore, ScO_2_ decrease during cardiopulmonary bypass (CPB) associates with increased postoperative multiorgan dysfunction syndrome [9]. During non-cardiac surgery, ScO_2_ monitoring could also minimize brain exposure to hypoxia in elderly patients [10].

Postoperative cognitive dysfunction (POCD) is a frequent complication in elderly patients undergoing major non-cardiac surgery [11, 12], and could be a manifestation of transient or permanent cerebral injury. The etiology of POCD is unclear but likely involves a combination of patient, surgical, and anesthetic factors. ScO_2_ decrease may associate with POCD, but previous studies commonly focused on cardiac surgery [13, 14], which means the ScO_2_ results could be affected by CPB. Total knee arthroplasty (TKA) is among the most common major non-cardiac surgeries performed on elderly population [15], and in previous studies with TKA, general anesthesia was commonly performed [16, 17], so the ScO_2_ data could be affected by mechanical ventilation and general anesthetics.

The present study was designed to elucidate the relationship between perioperative ScO_2_ and POCD in elderly patients undergoing TKA. The intrathecal anesthesia was selected to avoid the impact of mechanical ventilation and general anesthetics and provide the relatively physiological status of ScO_2_ in elderly patients. Furthermore, we observed the possible perioperative influential factors and their impacts on ScO_2_, and verified the hypothesis that ScO_2_ after multiple influential factors could predict the occurrence of POCD.

## Methods

The present study was approved by Peking university third hospital medical ethics committee (No. IRB00006761). Patients aged more than 65 years undergoing elective TKA between October 20, 2014 and March 31, 2015 were enrolled in study group, and written informed consent was obtained from each patient. Exclusion criterion included psychiatric disorder, central nervous system disease, carotid stenosis, history of craniotomy, use of sedatives or antipsychotics, drug or alcohol dependence, visual, auditory or motor disability, and preoperative mini-mental state examination (MMSE) score less than 24.

Neuropsychological tests were administered one day before and 6 days after surgery in study group by the same physician, to characterize postoperative cognitive dysfunction. The test battery encompassed MMSE, digital span test, word recognition memory test, digit symbol substitution test, trail making test A, stroop color word interference (part 3) and verbal fluency test, which primarily focused on learning, memory, attention, concentration and executive function.

Then 20 healthy subjects were recruited as control group that matched the age, gender and education level of study group. Cognitive functions were assessed two times with 7 days interval, and the standard deviation of baseline score were calculated. Learning effects, which exist in reduplicative neuropsychological tests [18], were also calculated as mean variation of the second assessments from baselines.

The patients in study group were divided into non-POCD and POCD groups according to the international study of postoperative cognitive dysfunction (ISPOCD) [11, 18]. Specifically, from each neuropsychological test score, baseline score and learning effect were subtracted, and then the difference was divided by the standard deviation of baseline score in control group. The magnitude of result was called Z score, and patients with at least two Z scores > 2 were assigned into POCD group.

In study group, a cannula was inserted in radial artery upon arrival at the operation room, then arterial blood pressure (BP), ECG, SpO_2_ and body temperature were monitored and maintained within physiological range. Spinal anesthesia was induced in L_2–3_ or L_3–4_ vertebra interspace, 0.15 mg/kg bupivacaine was administered with upper sensory blockade at T_8–10_. During anesthetic procedure, no sedative was provided. Arterial blood gas analysis results were recorded prior to bone cement implantation, and 5 min after. Fluid infusion and blood loss quantity, and surgical duration were also recorded. Percentage decreases of BP after anesthesia induction and tourniquet deflation, and PaO2 after bone cement implantation were calculated. After operation, continuous femoral nerve analgesia was performed.

Two sensors of FORE-SIGHT cerebral oximeter (near-infrared spectroscopy (NIRS) oximeter, CAS Medical Systems, Branford, CT) were placed on the left and right sides of the forehead for continuous ScO_2_ monitoring until the end of anesthesia. ScO_2_ was the average of left and right monitoring data. ScO_2_ before induction (T1), 10 min and 20 min after induction (T2 and T3), 10 min and 20 min after bone cement implantation (T4 and T5), and 10 min after tourniquet deflation (T6) were recorded respectively. Average ScO_2_ before induction (during 10 min) were regarded as the baseline value. Average ScO_2_ in 20 min after induction, in 20 min after bone cement implantation and in 10 min after tourniquet deflation were calculated, and their percentage decreases from baseline were calculated. Average ScO_2_ during anesthesia and minimum ScO_2_ were also recorded.

### Statistical analysis

As estimated with PASS (version 8.03, NCSS LLC, Kaysville, UT), a sample size of 70 patients would be sufficient to detect a difference in average ScO_2_ after multiple perioperative factors between non-POCD and POCD groups with a power of 0.9 and a significance level of 0.05. According to the criterion of ISPOCD, patients were divided into non-POCD and POCD groups. Continuous variables were expressed as mean ± SD and analyzed with paired *t*-test within group and independent *t*-test between groups. Categorical variables were expressed as numbers (percentages) and analyzed with chi-square test. ScO_2_ during operation was analyzed with two-way repeated-measures ANOVA. Receiver-operator characteristic (ROC) curves were generated to assess the predictive performance of ScO_2_ on the occurrence of POCD. Multivariate logistic regression model was used to determine independent risk factors of POCD. Data were analyzed with SPSS (version 21.0, IBM Corp, New York, NY). *P* < 0.05 was regarded as statistically significant.

## Results

A total of 80 patients were enrolled in the study. Two patients were excluded from the study, among which one failed to complete neuropsychological tests and the other experienced postoperative infection. As illustrated in Table 1, there was no difference between study and control groups in age, gender, BMI, education, drinking, smoking and baseline MMSE scores. Neuropsychological test results of control group are summarized in Table 2. According to the criterion of ISPOCD, POCD occurred in 15 patients (19.2 %). Table 3 illustrates demographic and clinical characteristics of non-POCD and POCD groups. With tourniquet inflation, intraoperative blood losses were less than 20 ml, and body temperature was maintained within physiological range. Thus, these data have not been shown. The age of POCD group is significantly higher than non-POCD group (*P* < 0.01), and there is no significant difference between two groups in other demographics, hemoglobin concentration, ASA classification, surgical duration and fluid transfusion.

**Table 1:** Demographic of control and study groups

	Study (*n* = 78)	Control (*n* = 20)
Age (years)	70.42 ± 3.76	69.90 ± 3.63
Gender (M/F)	36/42	9/11
BMI (kg/m^2^)	26.46 ± 3.17	25.89 ± 2.29
Eduction (Less than middle school/Middle school/More than middle school, %)	71.8/24.4/3.8	80.0/15.0/5.0
Drinking (%)	21.8	20.0
Smoking (%)	14.1	15.0
Baseline MMSE scores	28.03 ± 1.59	28.00 ± 1.69

**Table 2:** Neuropsychological test scores of control group on baseline and second assessment day (7 days later)

	Baseline (*n* = 20)	7 days later (*n* = 20)
MMSE	28.00 ± 1.69	28.70 ± 1.26
Digital span test	13.40 ± 2.28	13.75 ± 2.07
Word recognition memory test	2.27 ± 0.48	2.20 ± 0.59
Digit symbol substitution test	31.20 ± 4.53	31.40 ± 4.60
Trail making test A (s)	41.95 ± 6.44	41.65 ± 8.43
Stroop color word interference	38.35 ± 5.97	41.35 ± 5.88
Verbal fluency test	18.90 ± 3.16	18.95 ± 3.35

Digit span test score = forward score + backward score

**Table 3:** Demographic and clinical characteristics of non-POCD and POCD groups

	Non-POCD (*n* = 63)	POCD (*n* = 15)
Age (years)	69.75 ± 3.48	73.27 ± 3.67**
Gender (M/F)	28/34	8/7
BMI (kg/m^2^)	26.35 ± 3.31	26.95 ± 2.53
Eduction (Less than middle school/Middle school/More than middle school, %)	71.4/23.8/4.8	73.3/26.7/0
Drinking (%)	20.6	26.7
Smoking (%)	12.7	20
Hemoglobin (g/L)	130.67 ± 10.76	129.87 ± 10.15
ASA classification (I/II/III, %)	57.1/41.3/1.6	40.0/53.3/6.7
Surgical duration (min)	87.92 ± 10.09	89.20 ± 13.57
Fluid infusion (ml)	1350.79 ± 259.57	1440.00 ± 284.86

**P < 0.01 in comparison with non-POCD group

Neuropsychological test results of non-POCD and POCD groups are summarized in Table 4. Postoperative MMSE, digital span test, word recognition memory test, stroop color word interference (part 3) and verbal fluency test scores of POCD group were significantly lower than non-POCD group (*P* < 0.01), but there was no significant difference in digit symbol substitution test and trail making test A. Postoperative MMSE, digital span test, word recognition memory test, trail making test A, stroop color word interference (part 3) and verbal fluency test scores of POCD group were significantly lower than baseline in POCD group (*P* < 0.05 or *P* < 0.01), but there was no significant difference in digit symbol substitution test and in non-POCD group.

**Table 4:** Neuropsychological test scores of non-POCD and POCD groups before and 6 days after surgery

	Baseline		6 days after surgery	
	Non-POCD (*n* = 63)	POCD (*n* = 15)	Non-POCD (*n* = 63)	POCD (*n* = 15)
MMSE	28.10 ± 1.54	27.73 ± 1.79	27.51 ± 1.86	25.33 ± 1.54*^,^ ***
Digital span test	13.16 ± 2.82	12.93 ± 2.19	12.75 ± 2.38	10.13 ± 2.47*^,^ ***
Word recognition memory test	2.22 ± 0.48	2.38 ± 0.47	2.40 ± 0.48	3.16 ± 0.43*^,^***
Digit symbol substitution test	31.71 ± 4.07	31.47 ± 4.58	30.54 ± 4.98	27.60 ± 6.03
Trail making test A (s)	41.02 ± 4.57	40.93 ± 3.90	42.10 ± 5.29	45.20 ± 6.46**
Stroop color word interference	38.94 ± 4.04	38.80 ± 3.69	37.37 ± 5.40	30.47 ± 6.09*^,^ ***
Verbal fluency test	18.62 ± 2.96	18.20 ± 3.10	17.56 ± 3.17	12.80 ± 3.80*^,^ ***

*P < 0.01 in comparison with non-POCD group; **P < 0.05, ***P < 0.01 in comparison with baseline of either group. Digit span test = forward score + backward score

BP and PaO_2_ fluctuation of non-POCD group and POCD group are summarized in Table 5. Compared with baseline (before anesthesia induction and tourniquet deflation), BP after anesthesia induction and tourniquet deflation decreased significantly (*P* < 0.01), and the percentage decreases of POCD group were significantly higher than non-POCD group (*P* < 0.05 or *P* < 0.01). Compared with baseline (before cement implantation), PaO_2_ after cement implantation decreased significantly (*P* < 0.01), but there was no significant difference in percentage decrease between non-POCD and POCD groups.

**Table 5:** BP and PaO_2_ decrease of non-POCD and POCD groups

	Non-POCD (*n* = 63)		POCD (*n* = 15)	
	Baseline	After	Baseline	After
BP during anesthesia induction (mmHg)	98.70 ± 8.89	85.40 ± 7.32*	104.47 ± 8.76	82.93 ± 5.57*
BP decrease after anesthesia induction (%)	13.22 ± 6.21		22.24 ± 5.95***	
PaO_2_ during cement implantation (mmHg)	112.86 ± 9.51	107.78 ± 8.20*	111.60 ± 6.99	102.33 ± 6.50*
PaO_2_ decrease after cement implantation (%)	4.20 ± 6.85		8.06 ± 6.90	
BP during tourniquet deflation (mmHg)	95.14 ± 6.44	85.17 ± 7.16*	96.27 ± 5.92	82.73 ± 4.53*
BP decrease after tourniquet deflation (%)	10.56 ± 5.08		13.83 ± 5.90**	

*P < 0.01 in comparison with baseline of either group, **P < 0.05, ***P < 0.01 in comparison with non-POCD group of BP and PaO_2_ decrease

**Fig. 1:**
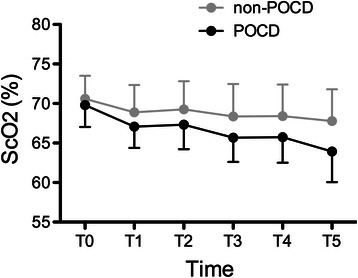
ScO_2_ during anesthesia of non-POCD and POCD groups. ScO_2_ decreased during anesthesia, and ScO_2_ of POCD group was significantly lower than non-POCD group (*P* < 0.05). T1-6: before induction, 10 min and 20 min after anesthesia induction, 10 min and 20 min after cement implantation, and 10 min after tourniquet deflation

**Fig. 2:**
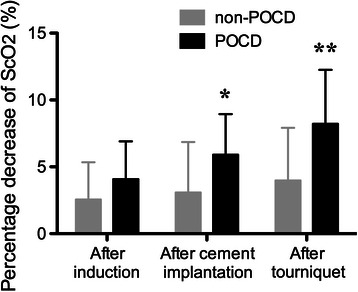
Percentage decreases of ScO_2_ in non-POCD and POCD groups after anesthesia induction, cement implantation and tourniquet deflation. *P < 0.05, **P < 0.01 in comparison with non-POCD group

Two-way repeated-measures ANOVA shows that ScO_2_ decreased significantly during anesthesia (*P* < 0.01). ScO_2_ of POCD group was significantly lower than non-POCD group (*P* < 0.05), and time × group interaction was significant (*P* < 0.01, Fig. 1). The average ScO_2_ after anesthesia induction, cement implantation and tourniquet deflation and their percentage decreases from baseline (before anesthesia induction) was calculated. There was no significant difference in baseline ScO_2_. After anesthesia induction, there was also no significant difference between two groups, but with accumulative effects of multiple influential factors, there were significant differences in absolute values and percentage decreases of ScO_2_ between two groups after cement implantation (*P* < 0.05) and tourniquet deflation (*P* < 0.01, Table 6 and Fig. 2). Besides, there was no significant difference in average ScO_2_ during anesthesia, and minimum ScO_2_ of POCD group was significantly lower than non-POCD group (*P* < 0.05, Table 6).

**Table 6:** Baseline, average and minimum ScO_2_ of non-POCD and POCD groups

	Non-POCD (*n* = 63)	POCD (*n* = 15)
Baseline ScO_2_ (%)	70.63 ± 3.01	69.67 ± 2.58
Average ScO_2_ after anesthesia induction (%)	68.79 ± 3.49	66.87 ± 2.72
Average ScO_2_ after cement implantation (%)	68.41 ± 3.95	65.60 ± 2.90*
Average ScO_2_ after tourniquet deflation (%)	67.78 ± 4.03	64.00 ± 3.78**
Average ScO_2_ (%)	68.67 ± 3.51	66.73 ± 2.74
Minimum ScO_2_ (%)	62.37 ± 3.64	60.07 ± 3.41*

*P < 0.05, **P < 0.01 in comparison with non-POCD group

**Fig. 3:**
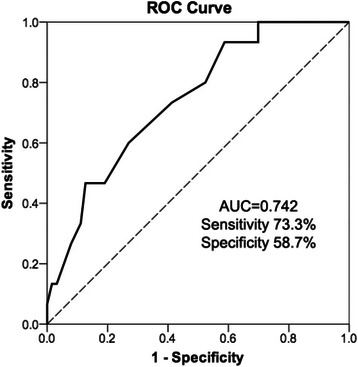
Receiver-operator characteristic (ROC) curve for average ScO_2_ after tourniquet deflation to predict the occurrence of POCD. The area under ROC curve is 0.742, which is significantly different from random chance (*P* < 0.01). The optimal threshold for POCD detection is 66.5 %, with sensitivity and specificity are 73.3 and 58.7 % respectively

There were significant differences in minimum ScO_2_, average ScO_2_ after cement implantation and tourniquet deflation between non-POCD and POCD groups. Therefore, ROC curves were generated to compare their predictive performances on the occurrence of POCD, and the areas under ROC curves (AUC) were 0.686 (*P* < 0.05), 0.707 (*P* < 0.05) and 0.742 (*P* < 0.01) respectively, which indicated average ScO_2_ after tourniquet deflation as the best predictor. Its optimal threshold for POCD detection was 66.5 %, with sensitivity and specificity were 73.3 and 58.7 % respectively (Fig. 3). The multivariate logistic regression determined that higher age (*P* < 0.05, OR = 1.221) and lower average ScO_2_ after tourniquet deflation (*P* < 0.05, OR = 0.835) were independent predictors of POCD.

## Discussion

The present results indicated that ScO_2_ of patients with POCD was lower than patients without POCD undergoing TKA with intrathecal anesthesia. Two previous studies on the relationship between ScO_2_ and POCD in TKA were performed with general anesthesia [16, 17], in which its ScO_2_ data could be affected by mechanical ventilation and general anesthetics, and was far from the physiological status of ScO_2_ in elderly patients. Another study on TKA with intrathecal anesthesia indicated that a trend of asymmetry in ScO_2_ could warn POCD [19]. Thus, we firstly revealed the relationship between perioperative ScO_2_ and the occurrence of POCD in TKA with intrathecal anesthesia.

TKA is among the most common major non-cardiac surgeries performed on the elderly population [15], and the prevalence of POCD after TKA is high, which has been reported as 19.4–72.0 % at one week and 6.5–29.5 % at 6 months postoperatively [20–22]. In the present study, POCD occurred in 19.2 % elderly patients undergoing TKA, and older patients had higher occurrence of POCD.

ScO_2_, estimated by NIRS light attenuation (wavelengths for oxyhemoglobin and deoxyhemoglobin) during cerebral tissue transmition [23], is a sensitive marker for cerebral hypoperfusion during major surgeries [24] and cardiac arrest [25, 26]. Low baseline and intraoperative ScO_2_ correlated with increased postoperative morbidity and mortality, as well as prolonged hospital stay [27, 28]. Factors affecting cerebral perfusion could result in ScO_2_ decrease [29], and correlation has been reported between ScO_2_ and transcranial Doppler, which indicates that ScO_2_ decrease could also predict cerebral ischemia [30, 31].

The etiology of cerebral oxygen desaturation is multifactorial, and the most commonly reported perioperative factor is embolism and hypoperfusion related to CPB [32]. In the present study, three influential factors including induction of spinal anesthesia, bone cement implantation and tourniquet deflation were observed. BP and ScO_2_ decreased after anesthesia induction and tourniquet deflation, and PaO_2_ and ScO_2_ decreased after cement implantation. Furthermore, with the accumulative effects of multiple influential factors, patients with POCD showed lower ScO_2_ and higher decrease percentage from baseline level, compared with patients without POCD.

Atallah et al. [33] observed cerebral perfusion decrease and oxygen desaturation after spinal anesthesia in TURP patients, and speculate that cerebral oxygen desaturation contribute to TURP syndrome including yawning, irritability and nausea. In the present study, spinal anesthesia could also contribute to ScO_2_ decrease. Bone cement implantation syndrome (BCIS) is originated from cement related embolization and anaphylaxis, and characterized by clinical features including hypoxia, hypotension, pulmonary pressure increase and cardiac arrest [34]. BCIS and related cerebral microembolism could emerge during TKA [35]. After tourniquet deflation, cement, fat and bone related embolization could also emerge [36]. In the present study, no typical BCIS was observed, but PaO_2_ decreased after cement implantation and BP decreased after tourniquet deflation, which might relate to multiple embolisms and contribute to ScO_2_ decrease.

As there was no difference in other influential factors including hemoglobin and temperature, we considered anesthesia induction, cement implantation and tourniquet deflation as major influential factors of ScO_2_ fluctuation during TKA. The strategies to treat ScO_2_ decrease during TKA could comprise head reposition, PaCO_2_ and BP elevation, vasodilation and blood transfusion, however, their effects need further investigations. During cardiac surgery, interventions also comprise perfusion cannulae and pump flow rate adjustment, temperature reduction and anesthetic depth adjustment, and these interventions could decrease the occurrence of POCD and length of hospital stay [37].

Fudickar et al. [38] use minimum ScO_2_ to predict the occurrence of POCD, but only found week predictive performance (AUC = 0.61), while we found that ScO_2_ after multiple influential factors had better discriminatory power for POCD. ScO_2_ less than 50 % or decrease 20 % from baseline during carotid endarterectomy indicated focal cerebral ischemia [39], and were commonly regarded as safe limits of ScO_2_ and chosen as the low limits in the present study. Once low ScO_2_ appeared, interventions including BP elevation and fluid infusion were performed, which could be responsible for the week predictive performance of minimum ScO_2_. As ScO_2_ after all influential factors had best predictive performance, its optimal threshold for POCD detection was determined. A relatively higher sensitivity was selected considering patients safety, and the result is that actively maintaining ScO_2_ after all influential factors above 66.5 % in elderly patients could decrease the prevalence of POCD.

Hypoxia could impair neuronal protein synthesis and synaptic plasticity [40], and link to learning and memory impairment [41]. Exposure to hypoxia triggers hypoxia-inducible factor (HIF) [42] and prolyl hydroxylases family [43], which affect cell survival. Effective hypoxia sensing are critical for cell and organ function, while HIF, endothelin-1, heme oxygenase and tyrosine hydroxylase all involve in the regulation of brain hypoxia [44]. As aging cells have decreased hypoxic response and cytoprotection including HIF response [45], elderly patients may be more susceptible to brain hypoxia. Considering the present results, as well as above-mentioned mechanisms, we infer that cerebral hypoxia and related mechanism are among the major mechanisms responsible for the occurrence of POCD.

Although ScO_2_ provided by NIRS has been demonstrated valuable during multiple circumstances, it still has limitations. Firstly, NIRS oximeters were put on forehead and reflected the condition of superficial cerebral cortex, so it might miss embolism, hypoperfusion and desaturation far from superficial cortex [46]. Secondly, Neurotoxicity such as neuroinflammation and Aβ generation also contribute to POCD [47–49], which affect the predictive performance of ScO_2_. Thirdly, even if absolute value of 50 and 80 % of baseline were regarded as safe limits for ScO_2_ to prevent cerebral ischemia, cognitive dysfunction were still observed in the present and previous studies [13]. Thus, these limits of ScO_2_ are not adequate, and our results indicated that 66.5 % could be a better limit for ScO_2_ to avoid POCD. Finally, we have not performed transesophageal echocardiographic assessment. As BCIS and other factors related cardiac dysfunction are not uncommon and could affect cerebral oxygen supply, echocardiography could be valuable in TKA and should be performed in the future study.

## Conclusion

The present study indicates that the incidence of POCD in elderly patients undergoing TKA with intrathecal anesthesia is 19.2 %. Perioperative ScO_2_ of patients with POCD is lower than patients without POCD, and ScO_2_ after multiple perioperative influential factors could be an effective predictor for the occurrence of POCD. These results reveal an important role of ScO_2_ decrease in the development of POCD, and provide a convenient monitoring method and possible treatment target.

## Abbreviations

ScO_2_: Cerebral oxygen saturation
POCD: Postoperative cognitive dysfunction
CPB: Cardiopulmonary bypass
TKA: Total knee arthroplasty
MMSE: Mini-mental state examination
ISPOCD: International study of postoperative cognitive dysfunction
ROC curve: Receiver-operator characteristic curve
AUC: Area under ROC curve
NIRS: Near-infrared spectroscopy
BCIS: Bone cement implantation syndrome
HIF: Hypoxia-inducible factor

## References

[1] Watzman HM, Kurth CD, Montenegro LM, Rome J, Steven JM, Nicolson SC (2000). Arterial and venous contributions to near-infrared cerebral oximetry. Anesthesiology.

[2] Colak Z, Borojevic M, Bogovic A, Ivancan V, Biocina B, Majeric-Kogler V (2015). Influence of intraoperative cerebral oximetry monitoring on neurocognitive function after coronary artery bypass surgery: a randomized, prospective studydaggerdouble dagger. Eur J Cardiothorac Surg.

[3] Yoshitani K, Kuwajima K, Irie T, Inatomi Y, Miyazaki A, Iihara K, Ohnishi Y (2013). Clinical validity of cerebral oxygen saturation measured by time-resolved spectroscopy during carotid endarterectomy. J Neurosurg Anesthesiol.

[4] Kishi K, Kawaguchi M, Yoshitani K, Nagahata T, Furuya H (2003). Influence of patient variables and sensor location on regional cerebral oxygen saturation measured by INVOS 4100 near-infrared spectrophotometers. J Neurosurg Anesthesiol.

[5] Teng Y, Ding H, Gong Q, Jia Z, Huang L (2006). Monitoring cerebral oxygen saturation during cardiopulmonary bypass using near-infrared spectroscopy: the relationships with body temperature and perfusion rate. J Biomed Opt.

[6] Faulkner JT, Hartley M, Tang A (2011). Using cerebral oximetry to prevent adverse outcomes during cardiac surgery. Perfusion.

[7] Sun X, Ellis J, Corso PJ, Hill PC, Lowery R, Chen F, Lindsay J (2014). Mortality predicted by preinduction cerebral oxygen saturation after cardiac operation. Ann Thorac Surg.

[8] Goldman S, Sutter F, Ferdinand F, Trace C (2004). Optimizing intraoperative cerebral oxygen delivery using noninvasive cerebral oximetry decreases the incidence of stroke for cardiac surgical patients. Heart Surg Forum.

[9] Murkin JM, Adams SJ, Novick RJ, Quantz M, Bainbridge D, Iglesias I, Cleland A, Schaefer B, Irwin B, Fox S (2007). Monitoring brain oxygen saturation during coronary bypass surgery: a randomized, prospective study. Anesth Analg.

[10] Casati A, Fanelli G, Pietropaoli P, Proietti R, Tufano R, Danelli G, Fierro G, De Cosmo G, Servillo G (2005). Continuous monitoring of cerebral oxygen saturation in elderly patients undergoing major abdominal surgery minimizes brain exposure to potential hypoxia. Anesth Analg.

[11] Moller JT, Cluitmans P, Rasmussen LS, Houx P, Rasmussen H, Canet J, Rabbitt P, Jolles J, Larsen K, Hanning CD (1998). Long-term postoperative cognitive dysfunction in the elderly ISPOCD1 study. ISPOCD investigators. International Study of Post-Operative Cognitive Dysfunction. Lancet.

[12] Monk TG, Weldon BC, Garvan CW, Dede DE, van der Aa MT, Heilman KM, Gravenstein JS (2008). Predictors of cognitive dysfunction after major noncardiac surgery. Anesthesiology.

[13] Yao FS, Tseng CC, Ho CY, Levin SK, Illner P (2004). Cerebral oxygen desaturation is associated with early postoperative neuropsychological dysfunction in patients undergoing cardiac surgery. J Cardiothorac Vasc Anesth.

[14] Zheng F, Sheinberg R, Yee MS, Ono M, Zheng Y, Hogue CW (2013). Cerebral near-infrared spectroscopy monitoring and neurologic outcomes in adult cardiac surgery patients: a systematic review. Anesth Analg.

[15] Harris WH, Sledge CB (1990). Total hip and total knee replacement (1). N Engl J Med.

[16] Papadopoulos G, Karanikolas M, Liarmakopoulou A, Papathanakos G, Korre M, Beris A (2012). Cerebral oximetry and cognitive dysfunction in elderly patients undergoing surgery for hip fractures: a prospective observational study. Open Orthopaedics J.

[17] Lin R, Zhang F, Xue Q, Yu B (2013). Accuracy of regional cerebral oxygen saturation in predicting postoperative cognitive dysfunction after total hip arthroplasty: regional cerebral oxygen saturation predicts POCD. J Arthroplasty.

[18] Rasmussen LS, Larsen K, Houx P, Skovgaard LT, Hanning CD, Moller JT, Dysfunction IgTISoPC (2001). The assessment of postoperative cognitive function. Acta Anaesthesiol Scand.

[19] Salazar F, Donate M, Boget T, Bogdanovich A, Basora M, Torres F, Gracia I, Fabregas N (2014). Relationship between intraoperative regional cerebral oxygen saturation trends and cognitive decline after total knee replacement: a post-hoc analysis. BMC Anesthesiol.

[20] Deo H, West G, Butcher C, Lewis P (2011). The prevalence of cognitive dysfunction after conventional and computer-assisted total knee replacement. Knee.

[21] Rodriguez RA, Tellier A, Grabowski J, Fazekas A, Turek M, Miller D, Wherrett C, Villeneuve PJ, Giachino A (2005). Cognitive dysfunction after total knee arthroplasty: effects of intraoperative cerebral embolization and postoperative complications. J Arthroplasty.

[22] Salazar F, Donate M, Boget T, Bogdanovich A, Basora M, Torres F, Fabregas N (2011). Intraoperative warming and post-operative cognitive dysfunction after total knee replacement. Acta Anaesthesiol Scand.

[23] Ferrari M, Mottola L, Quaresima V (2004). Principles, techniques, and limitations of near infrared spectroscopy. Can J Appl Physiol.

[24] Plachky J, Hofer S, Volkmann M, Martin E, Bardenheuer HJ, Weigand MA (2004). Regional cerebral oxygen saturation is a sensitive marker of cerebral hypoperfusion during orthotopic liver transplantation. Anesth Analg.

[25] Kamarainen A, Sainio M, Olkkola KT, Huhtala H, Tenhunen J, Hoppu S (2012). Quality controlled manual chest compressions and cerebral oxygenation during in-hospital cardiac arrest. Resuscitation.

[26] Ito N, Nanto S, Nagao K, Hatanaka T, Nishiyama K, Kai T (2012). Regional cerebral oxygen saturation on hospital arrival is a potential novel predictor of neurological outcomes at hospital discharge in patients with out-of-hospital cardiac arrest. Resuscitation.

[27] Edmonds HL, Ganzel BL, Austin EH (2004). Cerebral oximetry for cardiac and vascular surgery. Semin Cardiothorac Vasc Anesth.

[28] Ono M, Brady K, Easley RB, Brown C, Kraut M, Gottesman RF, Hogue CW (2014). Duration and magnitude of blood pressure below cerebral autoregulation threshold during cardiopulmonary bypass is associated with major morbidity and operative mortality. J Thorac Cardiovasc Surg.

[29] Godet G, Couaud A, Lucas A, Cardon A, Beloeil H, Ecoffey C (2013). Cerebral oxygen saturation is improved by xenon anaesthesia during carotid clamping. HSR Proceedings in Intensive Care & Cardiovascular Anesthesia.

[30] Murkin JM, Arango M (2009). Near-infrared spectroscopy as an index of brain and tissue oxygenation. Br J Anaesth.

[31] Samra SK, Dy EA, Welch K, Dorje P, Zelenock GB, Stanley JC (2000). Evaluation of a cerebral oximeter as a monitor of cerebral ischemia during carotid endarterectomy. Anesthesiology.

[32] Diegeler A, Hirsch R, Schneider F, Schilling LO, Falk V, Rauch T, Mohr FW (2000). Neuromonitoring and neurocognitive outcome in off-pump versus conventional coronary bypass operation. Ann Thorac Surg.

[33] Atallah MM, Hoeft A, El-Ghorouri MA, Hammouda GE, Saied MM (1998). Does spinal anesthesia affect cerebral oxygenation during transurethral prostatectomy?. Reg Anesth Pain Med.

[34] Donaldson AJ, Thomson HE, Harper NJ, Kenny NW (2009). Bone cement implantation syndrome. Br J Anaesth.

[35] Sulek CA, Davies LK, Enneking FK, Gearen PA, Lobato EB (1999). Cerebral microembolism diagnosed by transcranial Doppler during total knee arthroplasty: correlation with transesophageal echocardiography. Anesthesiology.

[36] Parmet JL, Horrow JC, Pharo G, Collins L, Berman AT, Rosenberg H (1995). The incidence of venous emboli during extramedullary guided total knee arthroplasty. Anesth Analg.

[37] Slater JP, Guarino T, Stack J, Vinod K, Bustami RT, Brown JM, Rodriguez AL, Magovern CJ, Zaubler T, Freundlich K (2009). Cerebral oxygen desaturation predicts cognitive decline and longer hospital stay after cardiac surgery. Ann Thorac Surg.

[38] Fudickar A, Peters S, Stapelfeldt C, Serocki G, Leiendecker J, Meybohm P, Steinfath M, Bein B (2011). Postoperative cognitive deficit after cardiopulmonary bypass with preserved cerebral oxygenation: a prospective observational pilot study. BMC Anesthesiol.

[39] Cho H, Nemoto EM, Yonas H, Balzer J, Sclabassi RJ (1998). Cerebral monitoring by means of oximetry and somatosensory evoked potentials during carotid endarterectomy. J Neurosurg.

[40] Payne RS, Goldbart A, Gozal D, Schurr A (2004). Effect of intermittent hypoxia on long-term potentiation in rat hippocampal slices. Brain Res.

[41] Wong KK, Grunstein RR, Bartlett DJ, Gordon E (2006). Brain function in obstructive sleep apnea: results from the Brain Resource International Database. J Integr Neurosci.

[42] Wang GL, Jiang BH, Rue EA, Semenza GL (1995). Hypoxia-inducible factor 1 is a basic-helix-loop-helix-PAS heterodimer regulated by cellular O2 tension. Proc Natl Acad Sci U S A.

[43] Kaelin WG, Ratcliffe PJ (2008). Oxygen sensing by metazoans: the central role of the HIF hydroxylase pathway. Mol Cell.

[44] Powell FL, Kim BC, Johnson SR, Fu Z (2009). Oxygen sensing in the brain--invited article. Adv Exp Med Biol.

[45] Ndubuizu OI, Chavez JC, LaManna JC (2009). Increased prolyl 4-hydroxylase expression and differential regulation of hypoxia-inducible factors in the aged rat brain. Am J Physiol Regul Integr Comp Physiol.

[46] Bokeriia LA, Golukhova EZ, Breskina NY, Polunina AG, Davydov DM, Begachev AV, Kazanovskaya SN (2007). Asymmetric cerebral embolic load and postoperative cognitive dysfunction in cardiac surgery. Cerebrovasc Dis.

[47] Shen X, Dong Y, Xu Z, Wang H, Miao C, Soriano SG, Sun D, Baxter MG, Zhang Y, Xie Z (2013). Selective anesthesia-induced neuroinflammation in developing mouse brain and cognitive impairment. Anesthesiology.

[48] Xie Z, Culley DJ, Dong Y, Zhang G, Zhang B, Moir RD, Frosch MP, Crosby G, Tanzi RE (2008). The common inhalation anesthetic isoflurane induces caspase activation and increases amyloid beta-protein level in vivo. Ann Neurol.

[49] Tao G, Zhang J, Zhang L, Dong Y, Yu B, Crosby G, Culley DJ, Zhang Y, Xie Z (2014). Sevoflurane induces tau phosphorylation and glycogen synthase kinase 3beta activation in young mice. Anesthesiology.

